# A decade of aging in healthy older adults: longitudinal findings on cerebrovascular and cognitive health

**DOI:** 10.1007/s11357-023-00790-w

**Published:** 2023-04-13

**Authors:** Ralf W. J. Weijs, Madelijn H. Oudegeest-Sander, Janneke I. A. Vloet, Maria T. E. Hopman, Jurgen A. H. R. Claassen, Dick H. J. Thijssen

**Affiliations:** 1 Department of Medical BioSciences, Radboud University Medical Center, Geert Grooteplein Zuid 10, 6525 GA Nijmegen, The Netherlands; 2 Department of Geriatrics, Radboudumc Alzheimer Center, Donders Institute for Brain, Cognition and Behaviour, Radboud University Medical Center, Nijmegen, The Netherlands; 3 Department of Cardiovascular Sciences, University of Leicester, Leicester, UK; 4 Research Institute for Sport and Exercise Sciences, Liverpool John Moores University, Liverpool, UK

**Keywords:** Aged, Brain blood flow, Cognition, Dementia, Longevity, Longitudinal studies

## Abstract

Research suggests an association between cerebrovascular health and cognitive decline, but previous work is limited by its cross-sectional nature or short (< 1–2 years) follow-up. Our aim was to examine, across 10 years of follow-up in healthy older adults, changes in cerebrovascular health and their relationship with subjective memory complaints as an early marker of cognitive decline. Between 2008 and 2010, twenty-eight healthy older adults (69 ± 4 years) underwent baseline blood pressure and transcranial Doppler measurements to assess middle cerebral artery blood velocity (MCAv), cerebrovascular resistance index (CVRi), and measures of cerebral autoregulation (CA). After 9–12 years of follow-up, these measurements were repeated, and presence of memory complaints was evaluated. Linear mixed-model analyses explored effects of aging on cerebrovascular parameters and whether memory complaints were associated with cerebrovascular changes. Across a median follow-up of 10.9 years, no changes in MCAv, CVRi, or CA were found. At baseline, these parameters were not different between subjects with (*n* = 15) versus without (*n* = 13) memory complaints. During follow-up, subjects with memory complaints showed larger decreases in MCAv (− 10% versus + 9%, *P* = 0.041) and increases in CVRi (+ 26% versus − 9%, *P* = 0.029) compared to other peers without memory complaints, but no distinct changes in CA parameters (*P* > 0.05). Although a decade of aging does not lead to deterioration in cerebral blood flow or autoregulation, our findings suggest that reductions in cerebral blood flow and increases in cerebrovascular resistance are associated with early subjective cognitive decline.

**Supplementary Information:**

The online version contains supplementary material available at 10.1007/s11357-023-00790-w.

## Introduction

Dementia, an umbrella term for neurodegenerative diseases, is strongly age-related and characterized by progressive cognitive decline [1]. Given the rising life expectancy of the global population, the worldwide prevalence of dementia is exponentially growing, causing a significant societal and economic burden [2]. The pathophysiology of dementia can span decades, covering the progression from asymptomatic to (subjective) cognitive impairment and ultimately dementia. Early cognitive symptoms (e.g., subjective complaints, objective episodic memory loss), that become more prevalent with aging, are associated with higher risk for future cognitive decline and/or dementia [3, 4]. Therefore, these early symptoms represent an opportunity for interventions aimed at prevention of dementia. It is imperative that we understand the underlying (patho)physiological processes of the early manifestation of cognitive decline.

Cerebrovascular pathology may play a major role in cognitive decline. In addition to ischemic neuronal damage resulting from stroke, animal models demonstrate that impaired neurovascular coupling, caused, for example, by endothelial or astrocytic dysfunction, underlies cognitive decline in aging [5, 6]. Previous cross-sectional studies demonstrated that individuals with mild cognitive impairment or dementia had lower cerebral blood flow (CBF) compared to cognitively healthy age-matched peers and that lower CBF was independently associated with progressive cognitive decline and dementia risk [7–9]. Moreover, our recent meta-analysis found a relation between reductions in CBF and cognitive decline in patients with dementia [10]. Specifically, reduction rates in CBF in these patients were ~ 3–10 times higher compared to healthy aging [10]. While this highlights the potential relation between cerebrovascular health and cognitive decline, the direction of this association remains uncertain. Therefore, long-term follow-up studies in older individuals who may ultimately develop cognitive decline are warranted.

Between 2008 and 2010, we performed comprehensive baseline measurements of CBF and autoregulation in healthy (i.e., normal cognitive function and free from chronic disease or significant morbidity) older adults. This provided a unique opportunity to perform a follow-up study in these same individuals to understand the impact of aging on cerebrovascular physiology and cognitive health. The primary aim of this study was to examine changes in cerebral hemodynamics and autoregulation in healthy older adults across 10-year follow-up. Previous work suggests that CBF, but not cerebral autoregulation, impairs with aging [11]. However, these studies are typically cross-sectional in nature [12–14]. Second, we explored whether (changes in) cerebral hemodynamics and autoregulation were related to development of subjective memory complaints, a prognostic marker for cognitive decline and dementia [3]. We hypothesized that across follow-up, healthy older adults show a marginal decline in CBF, while this effect is strongest in participants who develop memory complaints. Furthermore, we hypothesized that cerebral autoregulation is not affected by aging, irrespective of development of memory complaints.

## Methods

### Study design and participants

The population of interest for inclusion in this study comprised forty-eight community-dwelling older adults (age ≥ 65 years) who underwent baseline measurements as part of a previous study (ClinicalTrials.gov identifier: NCT01417663) [15]. Upon checking requested mortality data (Dutch Personal Records Database), we contacted all non-deceased individuals who previously consented to being contacted for future research, to gauge their interest to enroll in the present observational follow-up study. We requested for participation for repeated non-invasive measurements of cerebrovascular, cognitive, cardiorespiratory, and physical function. As baseline measurements were performed between 2008 and 2010, and follow-up measurements in 2020, this represented ~ 10-year follow-up. Upon inclusion in the previous study for baseline measurements, all participants were non-smoking and reported to perform exercise ≤ 1 h/week in the preceding 5–10 years. In addition, participants were free from diabetes mellitus, hypercholesterolemia, or (a history of) cardio- or cerebrovascular disease, did not use hormone replacement therapy or any medication interfering with the cardiovascular system, and were not regularly treated by a physician. Moreover, based on a medical screening by a geriatrician, all patients were cognitively healthy and did not use any psychotropic medication. Detailed information on inclusion criteria is described previously [15]. All individuals that participated in the previous baseline study were eligible for inclusion in the present follow-up study, irrespective of health status, as long as they possessed adequate visual and auditory acuity to allow for neuropsychological testing and the capacity to provide informed consent.

### Procedures

As part of participation by the same individuals in the previous baseline study and the present follow-up study with 9–12 years in-between, information on age and sex was collected, and height and weight were measured. All participants completed The Older Persons and Informal Caregivers Survey - Short Form (TOPICS-SF) [16] at follow-up to evaluate subjective functional health and well-being and to calculate a frailty index (range 0–1). As part of this questionnaire, participants were asked to indicate whether they experienced subjective memory complaints (yes/no).

Objective cognitive performance scores (range 0–30) were determined using the Mini-Mental State Examination (MMSE) [17] and the Montreal Cognitive Assessment (MoCA) [18] at follow-up that incorporate assessments of executive function, memory, attention, and verbal fluency. The order of administering the MMSE and MoCA was randomized between individuals. The MMSE was also administered at baseline.

During 5 min of seated rest, continuous transcranial Doppler (TCD) assessments were performed to measure mean bilateral blood velocities in the middle cerebral arteries (MCAv), a proxy for CBF, by placing two probes (2 MHz; DWL Multi-Dop at baseline, DWL DopplerBox at follow-up) over the temporal window which were fixed with a customized headband (Spencer Technologies, Seattle, WA). Simultaneously, control parameters were continuously measured, including heart rate using electrocardiography, blood pressure using finger photoplethysmography (Finapres Medical Systems, Amsterdam), and end-tidal carbon dioxide (EtCO_2_) using capnography (BIOPAC Systems, Goleta, CA). All data signals were captured at a sampling rate of 200 Hz using a data acquisition system (MP150, BIOPAC Systems, Goleta, CA). Protocols regarding data collection, processing, and analysis at follow-up were identical to baseline protocols [8] and in accordance with the CARNet white paper [19]. We derived resting EtCO_2_, mean arterial pressure (MAP), MCAv, cerebrovascular resistance index (CVRi = MAP/MCAv), and parameters of cerebral autoregulation (CA), i.e., gain, normalized gain, phase, and coherence over the low (0.07–0.20 Hz) and very low (0.02–0.07 Hz) frequency domains.

At follow-up, functional mobility and balance were assessed using the 30-s chair stand test (30CST) and the Timed Up & Go (TUG). In addition, maximal handgrip strength was assessed, a measure of upper limb strength and indicator of functional capacity with potential implications for predicting future cognitive changes [20]. For this assessment, participants were instructed to squeeze a hand-held dynamometer (MAP, KERN&SOHN GmbH) six times as powerful as possible, while the left and right hand were alternated (each three times) with resting periods of ≥ 1 min in-between. In addition, participants completed the Longitudinal Ageing Study Amsterdam Physical Activity Questionnaire (LAPAQ) at follow-up to assess a daily physical activity score. Finally, an incremental maximal bicycle ergometer test was performed to measure maximal oxygen uptake (VO_2_max) at baseline and after follow-up as described previously [15]. At follow-up, participants were allowed to perform a submaximal Ästrand test instead of the maximal test to estimate VO_2_max [21], for example, in case a participant did not feel comfortable to achieve maximal exertion.

### Statistical analysis

The statistical analyses were all performed in IBM SPSS (version 25.0). Continuous data were visually checked for normality. Normally distributed data are presented as mean with standard deviation (SD), whereas non-parametric data are presented as median with interquartile range (IQR). Categorical data are presented as frequency number with percentage. To address the primary objective related to the impact of aging across 10-year follow-up, we compared baseline and follow-up measures at group level using parametric paired t-tests or non-parametric Wilcoxon signed-rank tests for complete data sets. Given the significant number of missing data on cerebrovascular parameters and VO_2_max, which is usual in performing studies with TCD or (sub)maximal bicycle ergometer tests in older populations, we used linear-mixed model analyses to control for missing data. This approach also enabled correction for potential confounding factors, including baseline age, sex, and time-by-sex interaction. These analyses were performed with a random intercept and with time as a fixed factor (baseline/follow-up) to evaluate changes across follow-up at group level. Statistical significance was set at *P* < 0.05.

In addressing our second research objective related to potential differences at baseline and/or during follow-up between subjects with memory complaints versus those without, we performed parametric independent t-tests and non-parametric Mann–Whitney U tests for baseline comparison. To compare categorical variables between timepoints or groups, Fisher’s exact tests were used. Subsequently, linear mixed-model analyses were performed to examine whether changes across time interacted between subjects with versus without memory complaints, using two fixed factors: time (baseline/follow-up) and presence of memory complaints during follow-up (yes/no). All linear mixed-model analyses were performed with correction for baseline age, sex, and time-by-sex interaction. For analyses with cerebrovascular parameters as independent variable, additional analyses were performed with additional correction for anti-hypertensive medication use during follow-up (yes/no) and its interaction with time, as anti-hypertensive treatment potentially influences these parameters.

## Results

**Fig. 1:**
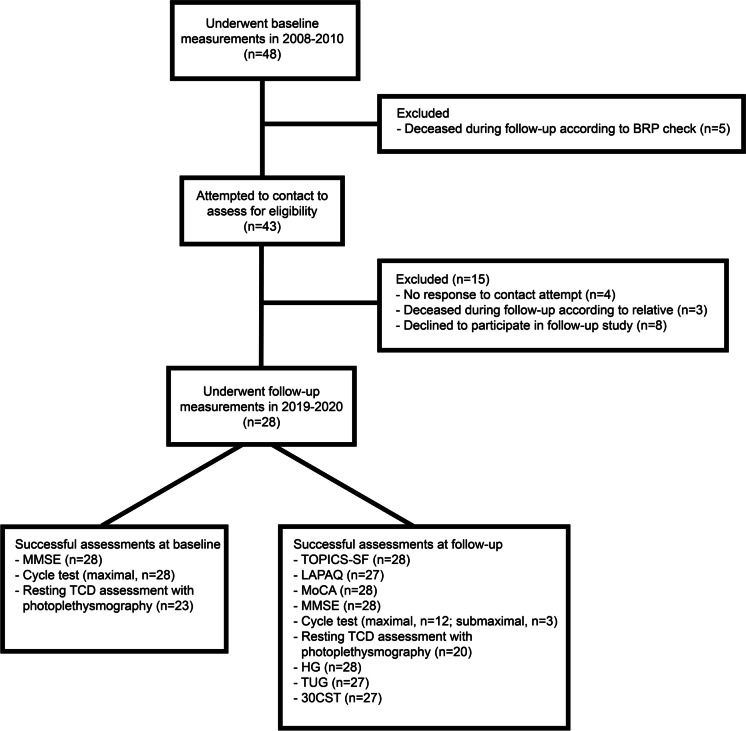
CONSORT-style flowchart diagram. Flowchart diagram showing the flow of participants from the original study population at baseline to inclusion in the present follow-up study, together with numbers of successful assessments for each type of measurement at both baseline and follow-up. Abbreviations: *30CST* 30-s chair stand test, *BRP* Dutch Personal Records Database, *HG* maximal handgrip strength, *LAPAQ* Longitudinal Ageing study Amsterdam Physical Activity Questionnaire, *MMSE* Mini-Mental State Examination, *MoCA* Montreal Cognitive Assessment, *TCD* transcranial Doppler, *TOPICS-SF* The Older Persons and Informal Caregivers Survey - Short Form, *TUG* Timed Up & Go. Adobe Illustrator (2021) was used to create Fig. 1

After a median follow-up duration of 131 (IQR 122–138) months, 28 out of 48 (58%) older adults were included in the present follow-up (aged 80.0 ± 3.5 years). A CONSORT-style flowchart can be found in Fig. 1.

At baseline, due to in- and exclusion criteria, subjects were healthy (i.e., free from morbidity), whereas at follow-up, all subjects reported (recent) presence of disease or complications during follow-up (Table 1). On the TOPICS-SF, 96% reported a score between 7 and 9 for subjective health, and 86% reported scores between 8 and 10 for quality of life. Across 10.9-year follow-up, BMI did not significantly change.

**Table 1:** Grouped data on demographic and physical characteristics at baseline and follow-up

Characteristic	Grouped (*N* = 28)		*P* value
	Baseline	Follow-up	
Age, years	69.1 ± 3.8	80.0 ± 3.5	** < 0.001**
Female vs male sex, *n* (%)	13 (46) vs 15 (54)		
BMI, kg/m^2^	26.3 ± 3.3	26.5 ± 4.4	0.83
MMSE, score 0–30	29.0 (28.0–30.0)	28.5 (27.0–29.0)	**0.035**
MoCA, score 0–30		25.0 (23.0–27.0)	
Level of education [22], *n* (%)			
Low, *n* (%)	3 (11)		
Middle,* n* (%)	11 (39)		
High, *n* (%)	14 (50)		
Presence of disease or complications during follow-up, *n* (%)			
Diabetes mellitus		0 (0)	
Anti-hypertensive medication use		13 (50)^a^	
Chronic obstructive pulmonary disease		3 (11)	
Arthritis		14 (50)	
Hearing impairment		14 (50)	
Visual impairment		5 (18)	
Dementia		0 (0)	
Neurological disease		0 (0)	
Transient ischemic attack		2 (7)	
Stroke		1 (4)	
Traumatic brain injury		1 (4)^a^	
Presence of disease or complications in 12 months preceding follow-up assessments, *n* (%)			
Heart disease		5 (18)	
Malignant form of cancer		2 (7)	
Depression		1 (4)	
Anxiety or panic attacks		2 (7)	
Hip fracture		0 (0)	
Dizziness		7 (25)	
Falls		7 (25)	
Subjective health, score 1–10		8 (7–8)	
Subjective quality of life, score 1–10		8 (8–9)	
Frailty index, score 0–1		0.09 (0.05–0.14)	
LAPAQ total daily physical activity score		89 ± 39^b^	
Maximal hand grip strength, kg		34 (24–43)	
Timed Up & Go, sec		8 (7–10)^b^	
30-s chair stand test, stands		11 ± 2^b^	

Data are presented as mean ± standard deviation, median with interquartile range between brackets, or frequency number with percentage between brackets. *P* values represent significance levels for comparisons between complete baseline and follow-up data using a paired t-test or Wilcoxon signed-rank test or for the fixed effect of time as derived from the linear mixed-model analyses with correction for baseline age, sex, and time-by-sex interaction. Values in bold indicate significance, *P* < 0.05

Abbreviations: *BMI* body mass index, *EtCO*_*2*_ end-tidal carbon dioxide, *LAPAQ* Longitudinal Ageing Study Amsterdam Physical Activity Questionnaire, *LF* low-frequency domain, *MMSE* Mini-Mental State Examination, *MoCA* Montreal Cognitive Assessment, *VLF* very low frequency domain, *VO*_*2*_*max* maximal oxygen uptake

^a^2 missing values

^b^1 missing value

Regarding cognitive function, we found a significant decrease in MMSE score by a median 0.5 point across follow-up (Table 1), while at both baseline and follow-up, none of the participants scored < 24, indicative of cognitive decline. The MoCA performance at follow-up revealed that twelve (43%) participants scored ≥ 26, which is considered normal. The remaining sixteen (57%) participants had MoCA scores between 20 and 25, that could possibly indicate mild cognitive impairment, of whom ten (63%) confirmed the presence of subjective memory complaints.

The incremental maximal bicycle ergometer test was successfully performed by all participants at baseline to assess VO_2_max. At follow-up however, this test was contraindicated in thirteen (46%) participants, while three (11%) participants completed a submaximal Ästrand test to estimate VO_2_max. Linear mixed-model analyses revealed no significant change in VO_2_max across follow-up (Table 2).

**Table 2:** Grouped cardiorespiratory fitness and cerebrovascular parameters at baseline and after follow-up

Parameter	Grouped (*N* = 28)						*P* value
	Baseline			Follow-up			
	Estimate	95% CI		Estimate	95% CI		
		Lower bound	Upper bound		Lower bound	Upper bound	
VO_2_max, ml/kg/min	24.7	23.3	26.2	22.5^a^	19.8	25.2	0.087
MAP, mmHg	88.5^b^	81.3	95.7	96.1^c^	86.6	105.6	0.15
MCAv, cm/s	49.1^b^	45.3	52.9	47.7^c^	43.9	51.5	0.50
CVRi, mmHg/cm/s	1.86^b^	1.67	2.04	2.08^c^	1.83	2.33	0.10
EtCO_2_, %	4.64^d^	4.34	4.93	4.49^d^	4.11	4.86	0.49
Gain_LF_, cm/s/mmHg	0.63^b^	0.54	0.71	0.63^d^	0.56	0.71	0.90
nGain_LF_, %/mmHg	1.29^b^	1.12	1.47	1.36^d^	1.21	1.51	0.54
Phase_LF_, degrees	32.2^b^	27.1	37.4	30.3^d^	22.5	38.0	0.66
Coherence_LF_, U	0.61^b^	0.52	0.70	0.66^c^	0.55	0.77	0.42
Gain_VLF_, cm/s/mmHg	0.50^b^	0.44	0.56	0.54^d^	0.43	0.64	0.53
nGain_VLF_, %/mmHg	1.03^b^	0.93	1.13	1.13^d^	0.93	1.34	0.31
Phase_VLF_, degrees	54.6^e^	48.4	60.9	49.3^d^	39.4	59.3	0.35
Coherence_VLF_, U	0.53^b^	0.47	0.59	0.60^c^	0.49	0.70	0.25

Data are presented as estimated marginal means with 95% CI. *P* values represent significance levels for the time effect derived from the linear mixed-model analyses

Abbreviations: *CVRi* cerebrovascular resistance index, *EtCO*_*2*_ end-tidal carbon dioxide, *LF* low-frequency domain, *MAP* mean arterial pressure, *MCAv* mean bilateral middle cerebral artery blood velocity, *nGain* normalized gain, *VLF* very low frequency domain, *VO*_*2*_*max* maximal oxygen uptake

^a^13 missing values

^b^5 missing values

^c^8 missing values

^d^9 missing values

^e^7 missing values

No significant effect of time on resting EtCO_2_, MAP, MCAv, CVRi, and CA parameters was found while correcting for baseline age, sex, and time-by-sex interaction (Table 2). A time effect was also absent with additional correction for anti-hypertensive treatment at follow-up and its interaction with time, although these additional analyses revealed significant time-by-treatment interaction effects on MAP (*P* < 0.001) and CVRi (*P* = 0.022).

Fifteen (54%) participants reported subjective memory complaints during follow-up. When comparing participants with versus without complaints (Table 3), groups were comparable in age at each timepoint. Sex proportions differed significantly, with relatively more females in the group with memory complaints. At baseline, participants with memory complaints had a lower BMI. Although groups similarly scored subjective health and quality of life at follow-up, participants with memory complaints had a higher frailty index as calculated based on answers to the TOPICS-SF.

**Table 3:** Demographic and physical characteristics for participants with and without subjective memory complaints

Characteristic	Subjective memory complaints		*P* value
	Yes (*n* = 15)	No (*n* = 13)	
Age at baseline, years	69.9 ± 4.3	68.2 ± 3.0	0.24
Age at follow-up, years	80.7 ± 3.9	79.1 ± 2.9	0.22
Follow-up duration, months	129 (122–138)	135 (126–139)	0.38
Female vs male sex, *n* (%)	10 (67) vs 5 (33)	3 (23) vs 10 (77)	**0.030**
BMI at baseline, kg/m^2^	25.3 ± 2.6	28.0 ± 3.7	**0.030**
BMI at follow-up, kg/m^2^	25.4 ± 3.9	27.9 ± 4.8^a^	0.17
MMSE at baseline, score 0–30	29.0 (28.0–29.0)	29.0 (28.0–30.0)	0.35
MMSE at follow-up, score 0–30	28.0 (26.0–29.0)	29.0 (27.5–30.0)	0.42
Change in MMSE across follow-up, score	− 1.0 (− 2.0–0.0)	− 1.0 (− 1.0–0.5)	0.72
MoCA at follow-up, score 0–30	25.0 (23.0–27.0)	26.0 (23.5–28.5)	0.32
Level of education [22]			0.23
Low, *n* (%)	3 (20)	0 (0)	
Middle, *n* (%)	5 (33)	6 (46)	
High, *n* (%)	7 (47)	7 (54)	
Subjective health at follow-up, score 1–10	8 (7–8)	8 (7–8)	0.75
Subjective quality of life at follow-up, score 1–10	8 (7–8)	8 (8–9)	0.12
Frailty index at follow-up, score 0–1	0.12 ± 0.06	0.07 ± 0.04	**0.015**
Anti-hypertensive medication use at follow-up, *n* (%)	5 (33)	8 (73)^a^	0.11
LAPAQ total daily physical activity score at follow-up	93 ± 49	83 ± 23^b^	0.47
Maximal hand grip strength at follow-up, kg	28.2 ± 6.9	39.9 ± 11.5	**0.003**
Timed Up & Go at follow-up, sec	8.6 ± 1.5	8.0 ± 1.5^b^	0.30
30-s chair stand test at follow-up, stands	11.1 ± 2.1	11.3 ± 2.0^b^	0.74
VO_2_max at baseline, ml/kg/min	24.8 ± 3.7	24.9 ± 4.5	0.96
MAP at baseline, mmHg	85.6 ± 13.1^a^	92.2 ± 18.9^c^	0.33
MCAv at baseline, cm/s	50.2 ± 9.6^a^	47.5 ± 7.7^c^	0.48
CVRi at baseline, mmHg/cm/s	1.77 ± 0.41^a^	1.98 ± 0.44^c^	0.24
EtCO_2_ at baseline, %	4.6 ± 0.6^d^	4.7 ± 0.6^e^	0.83
Gain_LF_ at baseline, cm/s/mmHg	0.65 ± 0.18^a^	0.60 ± 0.21^c^	0.50
nGain_LF_ at baseline, %/mmHg	1.33 ± 0.37^a^	1.25 ± 0.39^c^	0.64
Phase_LF_ at baseline, degrees	32.3 ± 12.2^a^	32.3 ± 10.9^c^	1.0
Coherence_LF_ at baseline, U	0.61 ± 0.22^a^	0.61 ± 0.16^c^	0.98
Gain_VLF_ at baseline, cm/s/mmHg	0.51 ± 0.13^a^	0.50 ± 0.12^c^	0.81
nGain_VLF_ at baseline, %/mmHg	1.04 ± 0.28^a^	1.05 ± 0.20^c^	0.90
Phase_VLF_ at baseline, degrees	54.0 ± 17.9^e^	51.5 ± 11.6^c^	0.72
Coherence_VLF_ at baseline, U	0.53 ± 0.17^a^	0.54 ± 0.11^c^	0.94

Data are presented as mean ± standard deviation, median with interquartile range between brackets, or frequency number with percentage between brackets. *P* values represent significance levels for comparisons between participants with versus without subjective memory complaints using independent t-tests and non-parametric Mann–Whitney U tests. Values in bold indicate significance, *P* < 0.05

Abbreviations: *BMI* body mass index, *EtCO*_*2*_ end-tidal carbon dioxide, *LAPAQ* Longitudinal Ageing Study Amsterdam Physical Activity Questionnaire, *LF* low-frequency domain, *MAP* mean arterial pressure, *MCAv* mean bilateral middle cerebral artery blood velocity, *MMSE* Mini-Mental State Examination, *MoCA* Montreal Cognitive Assessment, *nGain* normalized gain, *VLF* very low frequency domain, *VO*_*2*_*max* maximal oxygen uptake

^a^2 missing values

^b^1 missing value

^c^3 missing values

^d^5 missing values

^e^4 missing values

Distributions on educational level did not differ between participants with versus without memory complaints. No group differences were found in MMSE at baseline or follow-up, neither in MoCA at follow-up, and changes in MMSE across follow-up were comparable.

Total physical activity and function (LAPAQ, TUG, and 30CST) were comparable between groups. Maximal handgrip strength was 1.4 times stronger in the group without memory complaints, without accounting for sex and/or age. While baseline VO_2_max was comparable, changes across follow-up differed between groups, i.e., − 27% versus + 6% (*P* = 0.010) for participants with versus without memory complaints, respectively (Table S1).

**Fig. 2:**
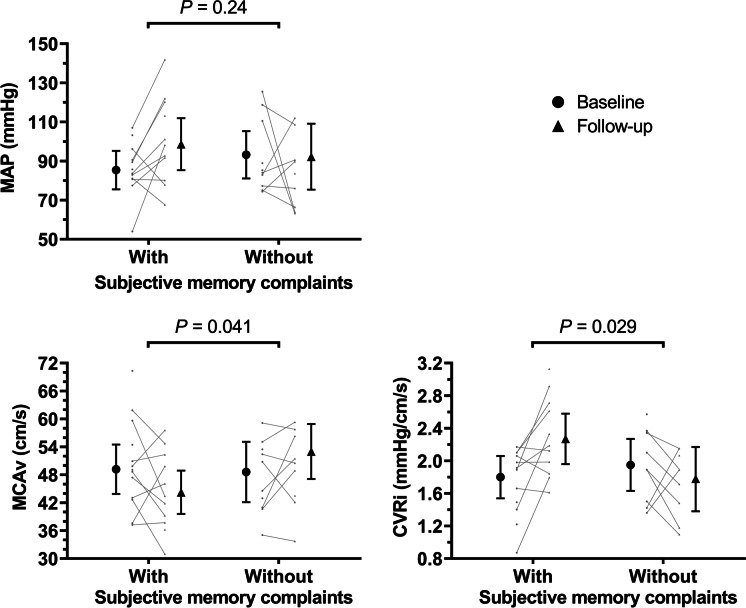
Longitudinal changes in cerebrovascular parameters in participants with and without subjective memory complaints. Graphs show estimated marginal means with 95% CI for MAP, MCAv, and CVRi at baseline (black circles) and after follow-up (black triangles) in participants with and without subjective memory complaints. Small gray data points show individual values available at each timepoint, with connecting lines indicating changes within participants. *P* values represent significance levels for the time-by-memory complaints interaction effect derived from the linear mixed-model analyses. Abbreviations: *CVRi* cerebrovascular resistance index, *MAP* mean arterial pressure, *MCAv* mean bilateral middle cerebral artery blood velocity. GraphPad Prism (version 9.3.1) was used to create Fig. 2

Groups with and without memory complaints had comparable baseline values of MAP, MCAv, CVRi, and CA (Table 3). Linear mixed-model analyses (Fig. 2; Table S1) revealed no significant time-by-memory complaints interaction effect for MAP, while participants with versus without complaints demonstrated significant opposite changes in MCAv, i.e., − 10% versus + 9% (*P* = 0.041), and CVRi, i.e. + 26% versus − 9% (*P* = 0.029). For all CA parameters, no differences in changes across follow-up were found between groups (Table S1).

## Discussion

Our study, aimed at evaluating the impact of a decade of aging in healthy older adults, presents the following observations. First, we found no significant changes in cerebrovascular parameters across 10.9-year follow-up at group level. Second, participants who reported subjective memory complaints during follow-up demonstrated a larger decrease in MCAv and increase in CVRi across follow-up compared to those without complaints, while groups revealed no differences in changes in CA. Third, we found no significant differences in cerebrovascular parameters between groups at baseline. Taken together, our long-term, within-subject follow-up suggests a relationship between reductions in CBF and increases in cerebrovascular resistance versus development of subjective memory complaints, which is of special relevance as this may represent an early marker of cognitive decline in a pre-clinical stage.

A unique aspect of our study is the 10.9-year follow-up, representing the longest follow-up of detailed vascular physiology in this age group to our knowledge. We found that a decade of healthy aging from ~ 70 to ~ 80 years old did not change cerebral autoregulation, which is important as most previous studies that examined the impact of age on CA adopted a cross-sectional design. These studies typically found preserved CA in older adults [23–25], with only one study reporting elevated transfer function gain (but not phase) in the oldest participants [26]. To our knowledge, only one other study adopted longitudinal follow-up and found a decrease in autoregulation index in ten middle-aged subjects (24–51 years at baseline) across 10-year follow-up [27]. Importantly, the autoregulation index after 10-year follow-up in this latter study still fell within normal ranges, and results on transfer function phase and gain were mixed [27]. Our study provides evidence that cerebral autoregulation does not deteriorate across 10.9-year follow-up in healthy older adults.

Previous studies found a small decrease in CBF with age, even in healthy individuals [12–14]. As these studies were cross-sectional, they were likely subject to sources of bias related to cohort effects, lifestyle-related factors, and/or (asymptomatic) disease. Nonetheless, we should acknowledge that our longitudinal study might be underpowered to detect small age-related changes. Previous cross-sectional studies estimate CBF reductions of ~ 0.5–1% annually [12–14], meaning that, with a baseline MCAv of 49.0 cm/s in our group, a decline of ~ 2.7–5.3 cm/s could have been expected across 10.9-year follow-up. We found a non-significant decline of 2.3 cm/s, which is slightly below these estimates. Therefore, our data seem supportive of a small age-related decline in CBF.

After follow-up, we divided groups based on the presence of subjective memory complaints, a proxy for higher risk of developing future cognitive decline and/or dementia [3]. We did not rely on cognitive screening tests (i.e., MMSE and/or MoCA) as changes in scores in the upper ranges, as mostly achieved by our participants, are less informative. Although a significant 0.5 median decrease in MMSE was found, the clinical relevance of this observation is questionable. To support this, we found no differences in MMSE or MoCA between groups with and without subjective memory complaints. While baseline values were comparable between groups, individuals with memory complaints demonstrated significantly larger decreases in MCAv (− 10% versus + 8%) and increases in CVRi (+ 26% versus − 7%). These observations reinforce our recent meta-analysis in patients with Alzheimer’s disease, wherein data from longitudinal studies showed that a higher annual decrease in CBF is associated with cognitive decline [10]. Cross-sectional studies reported that patients with mild cognitive impairment or dementia have lower CBF than healthy age-matched individuals [7–9, 28]. Our findings suggest that these cerebrovascular changes may already occur in early pre-clinical stages of cognitive decline. Supporting this hypothesis, previous work demonstrated that CVRi was elevated not only in Alzheimer’s disease but also in those in a prodromal stage of Alzheimer’s disease [28]. That study suggested that changes in cerebral hemodynamics may act as a pre-clinical biomarker for Alzheimer’s disease. In addition, a multifactorial data-driven analysis revealed that with aging, vascular dysregulation seems the earliest event in Alzheimer’s disease progression, even preceding amyloid deposition [29]. Unlike these previous studies, our study recruited community-dwelling healthy older adults, therefore avoiding selection bias of participants prone to dementia. Therefore, our data suggest that progressive changes in cerebral hemodynamics occur in, and may even precede, development of the earliest cognitive symptoms.

In contrast to the changes in CBF and cerebrovascular resistance, we found no differences in CA between groups with and without subjective memory complaints. Although impaired CA has been commonly hypothesized in cognitive decline or dementia, the majority of recent studies could not confirm that hypothesis [11]. Accordingly, our findings revealed that, even across long-term follow-up, CA did not change differently between subjects with versus without memory complaints. This means that the reduction in CBF in those with memory complaints cannot be explained by impaired CA. Equally, it is unlikely that it is caused by a change in MCA diameter. While changes in diameter affect MCAv, a lower MCAv can only be explained by vasodilation in the MCA. With the higher MAP in these participants, if anything, vasoconstriction of the MCA would be expected, not vasodilation.

Our observations raise the question on the potential mechanisms. First, previous studies demonstrated a potential link between cerebral atrophy and lower CBF; both processes have been linked to cognitive decline [10]. Whether cerebral atrophy precedes decreases in CBF or vice versa remains a topic of debate. Brain atrophy in neurodegenerative disease could reduce metabolic demand and thereby demand for CBF [10]. Vice versa, reduced oxygen and nutrient delivery by CBF can cause downstream atrophy. An alternative cause for reduced CBF may be cerebrovascular endothelial dysfunction, or astrocyte or pericyte dysfunction, hence a lower ability to respond to changes in neurological demand for oxygen and nutrients, i.e., neurovascular coupling [5, 6]. Subsequently, insufficient blood supply occurs causing hypoxia and neuronal damage. Future studies are required to further explore the potential underlying mechanisms.

The larger cerebral hemodynamic changes in individuals with early cognitive symptoms may be relevant for clinical management. Monitoring (proxies for) CBF has clinical potential in the early detection and/or prevention of cognitive decline. Another remarkable observation was that individuals with subjective memory complaints show marked worsening of cardiorespiratory fitness. This is in line with previous studies that showed that cardiorespiratory fitness, an indirect marker for cardiovascular health, is related to cognitive health [30, 31]. Interestingly, previous studies have linked regular exercise to improved cognitive function and reduced dementia incidence [32, 33], but also improved cerebral hemodynamics [11]. This provides further support for promotion of exercise and physical activity to prevent cognitive decline.

A few limitations of this study should be acknowledged. First, measurements of cerebrovascular parameters are subject to biological and technical measurement variation. This makes it difficult to detect small effect sizes, including the small MCAv reductions with aging. Second, we should stress that our results are possibly biased due to survivorship bias. Third, CA was only assessed under resting conditions. Therefore, our findings regarding CA may not be extrapolated to static CA (slow and large changes in blood pressure, e.g., due to anti-hypertensive treatment) or to dynamic CA under response to repeated orthostatic challenges. Finally, we found that subjects with subjective memory complaints were more frequently female and were more frail. However, our analyses corrected for sex, and we believe that frailty is inevitably tied to (early) cognitive decline and reductions in cardiorespiratory fitness. Therefore, this did not impact our main findings.

## Conclusion

In conclusion, our unique long-term follow-up study revealed that across 10.9-year follow-up, although grouped cerebral hemodynamics and autoregulation did not change in healthy older adults, larger decreases in MCAv and increases in CVRi were related to development of memory complaints. This is clinically relevant as early detection and prevention of cerebrovascular dysfunction, that is linked to cognitive decline, seem feasible by monitoring and targeting cardio- and cerebrovascular health. We warrant future studies to explore the causal links between potential underlying mechanisms, such as cerebral atrophy, and CBF reductions during aging.

### Supplementary Information

Below is the link to the electronic supplementary material.

Supplementary file1 (DOCX 18 KB)

## Data Availability

The data underlying this article will be shared on reasonable request to the corresponding author.
