# Supplementary material for: A decade of aging in healthy older adults: longitudinal findings on cerebrovascular and cognitive health
Source: GeroScience. 2023 Apr 13;45(4):2629–41. doi: 10.1007/s11357-023-00790-w (PMC10651595; doi:10.1007/s11357-023-00790-w)
Supplement: Supplementary file 1 — Supplementary file1 (DOCX 18 KB) [file 11357_2023_790_MOESM1_ESM.docx]

**Table S1. Cardiorespiratory fitness and cerebrovascular parameters at baseline and after follow-up for subjects with and without subjective memory complaints.**

| Parameter | Subjective memory complaints (n=15) | | | | | | No subjective memory complaints (n=13) | | | | | | P value |
| --- | --- | --- | --- | --- | --- | --- | --- | --- | --- | --- | --- | --- | --- |
|  | Baseline | | | Follow-up | | | Baseline | | | Follow-up | | |  |
|  | Estimate | 95% CI | | Estimate | 95% CI | | Estimate | 95% CI | | Estimate | 95% CI | |  |
|  |  | Lower  bound | Upper  bound |  | Lower  bound | Upper  bound |  | Lower  bound | Upper  bound |  | Lower  bound | Upper  bound |  |
| VO_2_max, ml/kg/min | 25.7 | 23.6 | 27.8 | 20.1 | 16.7 | 23.5 | 23.6 | 21.3 | 25.9 | 25.4 | 21.5 | 29.2 | **0.010** |
| MAP, mmHg | 85.4 | 75.5 | 95.2 | 98.6 | 85.3 | 111.9 | 93.2 | 81.1 | 105.3 | 92.2 | 75.4 | 109.1 | 0.24 |
| MCAv, cm/s | 49.2 | 43.9 | 54.5 | 44.2 | 39.6 | 48.9 | 48.6 | 42.1 | 55.1 | 53.0 | 47.1 | 58.9 | **0.041^a^** |
| CVRi, mmHg/cm/s | 1.80 | 1.54 | 2.06 | 2.27 | 1.96 | 2.58 | 1.95 | 1.63 | 2.27 | 1.78 | 1.38 | 2.17 | **0.029^b^** |
| EtCO_2_, % | 4.53 | 4.11 | 4.96 | 4.22 | 3.73 | 4.70 | 4.77 | 4.30 | 5.25 | 4.90 | 4.27 | 5.53 | 0.37 |
| Gain_LF_, cm/s/mmHg | 0.65 | 0.53 | 0.77 | 0.58 | 0.48 | 0.68 | 0.59 | 0.44 | 0.73 | 0.71 | 0.58 | 0.83 | 0.10 |
| nGain_LF_, %/mmHg | 1.34 | 1.10 | 1.58 | 1.38 | 1.16 | 1.60 | 1.22 | 0.93 | 0.52 | 1.33 | 1.07 | 1.60 | 0.78 |
| Phase_LF_, degrees | 32.6 | 25.4 | 39.7 | 32.4 | 21.6 | 43.3 | 31.7 | 22.8 | 40.5 | 27.1 | 13.8 | 40.5 | 0.67 |
| Coherence_LF_, U | 0.61 | 0.48 | 0.73 | 0.68 | 0.53 | 0.83 | 0.61 | 0.46 | 0.77 | 0.64 | 0.44 | 0.83 | 0.75 |
| Gain_VLF_, cm/s/mmHg | 0.52 | 0.45 | 0.60 | 0.47 | 0.34 | 0.61 | 0.47 | 0.37 | 0.56 | 0.63 | 0.46 | 0.79 | 0.10 |
| nGain_VLF_, %/mmHg | 1.08 | 0.94 | 1.21 | 1.07 | 0.80 | 1.35 | 0.96 | 0.79 | 1.13 | 1.23 | 0.89 | 1.57 | 0.23 |
| Phase_VLF_, degrees | 53.2 | 44.5 | 61.9 | 52.6 | 38.8 | 66.5 | 56.5 | 46.4 | 66.6 | 44.6 | 27.5 | 61.6 | 0.37 |
| Coherence_VLF_, U | 0.55 | 0.47 | 0.63 | 0.59 | 0.45 | 0.74 | 0.50 | 0.40 | 0.60 | 0.60 | 0.42 | 0.79 | 0.67 |

Data are presented as estimated marginal means with 95% CI. P-values represent significance levels for the time-by-memory complaints interaction effect derived from the linear mixed-model analyses.

Abbreviations: CVRi, cerebrovascular resistance index; EtCO_2_, end-tidal carbon dioxide; LF, low frequency domain; MAP, mean arterial pressure; MCAv, mean bilateral middle cerebral artery blood velocity; nGain, normalised gain; VLF, very low frequency domain; VO_2_max, maximal oxygen uptake.

^a^With additional correction for anti-hypertensive medication use, p=0.063.

^b^With additional correction for anti-hypertensive medication use, p=0.079.
